# Managing the link and strengthening transition from child to adult mental health Care in Europe (MILESTONE): background, rationale and methodology

**DOI:** 10.1186/s12888-018-1758-z

**Published:** 2018-06-04

**Authors:** H. Tuomainen, U. Schulze, J. Warwick, M. Paul, G.C. Dieleman, T. Franić, J. Madan, A. Maras, F. McNicholas, D. Purper-Ouakil, P. Santosh, G. Signorini, C. Street, S. Tremmery, F.C. Verhulst, D. Wolke, S. P. Singh, for the MILESTONE consortium, Swaran Singh, Helena Tuomainen, Jason Madan, Moli Paul, Cathy Street, Dieter Wolke, Jane Warwick, Priya Tah, Alastair Canaway, James Griffin, Philip Wells, Rebecca Appleton, Rose-Marie Lomax, Amanda Tuffrey, Anna Wilson, Charlotte Gatherer, Leanne Walker, Giovanni de Girolamo, Giulia Signorini, Giovanni Allibrio, Marco Armando, Stefano Vicari, Angelo Bertani, Giuseppe Carrà, Patrizia Conti, Francesco Margari, Ottaviano Martinelli, Emiliano Monzani, Francesco Rinaldi, Paolo Stagi, Paolo Scocco, Fabio Vanni, Alessandro Ferrari, Elisa Gheza, Cecilia Ferrari, Laura Rivolta, Flavia Levi, Maria Cataldo, Lidia Manenti, Giorgia Morini, Adriana Pastore, Cecilia Toselli, Pamela Varvara, Paramala Santosh, Natalie Heaney, Ilyas Sagar-Ouriaghli, Mathilde Mastroianni, Jatinder Singh, Diane Purper-Ouakil, Frédérick Russet, Virginie Maurice, Véronique Humbertclaude, Athanasios Maras, Larissa van Bodegom, Mathilde Overbeek, Arthur Van Gool, Therese van Amersfoort, Ulrike Schulze, Jörg M. Fegert, Paul Plener, Melanie Saam, Ulrike Breuninger, Anne Sartor, Elena Tanase, Renate Schepker, Michele Noterdaeme, Sabine Tremmery, Gaëlle Hendrickx, Fiona McNicholas, Aleksandra Gronostaj, Ingrid Holme, Lesley O’Hara, Ana Paula Paes de Mello de Camargo, Tomislav Franić, Nikolina Davidović, Paramala Santosh, Kate Lievesley, Federico Fiori, Frank Verhulst, Gwen Dieleman, Suzanne Gerritsen, Andrea Wohner, Sarah Buttle, Meghan Killilea, Courtney Smyth, Caoimhe Kelly, James Kirwin

**Affiliations:** 1 Mental Health and Wellbeing, Division of Health Sciences, Warwick Medical School, University of Warwick, Coventry, UK; 2 Department of Child and Adolescent Psychiatry/Psychotherapy, University of Ulm, Ulm, Germany; 3 Warwick Clinical Trials Unit, Warwick Medical School, University of Warwick, Coventry, UK; 4 Coventry and Warwickshire Partnership NHS Trust, Coventry, UK; 5 Department of Child and Adolescent Psychiatry and Psychology, Erasmus Medical Center, Rotterdam, Netherlands; 6 Department of Psychiatry, Clinical Hospital Center Split, Split, Croatia; 7 Warwick Medical School, University of Warwick, Coventry, UK; 8 Yulius Academy, Yulius Mental Health Organization, Barendrecht, Netherlands; 9 Department of Child and Adolescent Psychiatry, University College Dublin School of Medicine and Medical Science, Dublin, Republic of Ireland; 10 Geary Institute, University College Dublin, Dublin, Republic of Ireland; 11 Centre Hospitalier Universitaire de Montpellier, Montpellier, France; 12 Department of Child and Adolescent Psychiatry, Institute of Psychiatry, Psychology and Neuroscience, King’s College London, London, UK; 13 HealthTracker Ltd, Gillingham, UK; 14 Psychiatric Epidemiology and Evaluation Unit, Saint John of God Clinical Research Center, Brescia, Italy; 15 Department of Neurosciences, Child & Adolescent Psychiatry, University of Leuven, Leuven, Belgium; 16 Department of Child & Adolescent Psychiatry, University Hospitals Leuven, Leuven, Belgium; 17 Department of Psychology, University of Warwick, Coventry, UK; 18 Department of Child Psychiatry, Our Lady’s Hospital for Sick Children, Dublin, Republic of Ireland; 19 Lucena Clinic SJOG, Dublin, Republic of Ireland; 20 Centre for Interventional Paediatric Psychopharmacology and Rare Diseases (CIPPRD), National and Specialist Child and Adolescent Mental Health Services, Maudsley Hospital, London, UK

**Keywords:** Mental health, Child and adolescent mental health services, Transition, Health services research, Cluster randomised controlled trial, Longitudinal cohort study, Youth mental health, Policy, Professional training, Europe

## Abstract

**Background:**

Transition from distinct Child and Adolescent Mental Health (CAMHS) to Adult Mental Health Services (AMHS) is beset with multitude of problems affecting continuity of care for young people with mental health needs. Transition-related discontinuity of care is a major health, socioeconomic and societal challenge globally. The overall aim of the Managing the Link and Strengthening Transition from Child to Adult Mental Health Care in Europe (MILESTONE) project (2014–19) is to improve transition from CAMHS to AMHS in diverse healthcare settings across Europe. MILESTONE focuses on current service provision in Europe, new transition-related measures, long term outcomes of young people leaving CAMHS, improving transitional care through ‘managed transition’, ethics of transitioning and the training of health care professionals.

**Methods:**

Data will be collected via systematic literature reviews, pan-European surveys, and focus groups with service providers, users and carers, and members of youth advocacy and mental health advocacy groups. A prospective cohort study will be conducted with a nested cluster randomised controlled trial in eight European Union (EU) countries (Belgium, Croatia, France, Germany, Ireland, Italy, Netherlands, UK) involving over 1000 CAMHS users, their carers, and clinicians.

**Discussion:**

Improving transitional care can facilitate not only recovery but also mental health promotion and mental illness prevention for young people. MILESTONE will provide evidence of the organisational structures and processes influencing transition at the service interface across differing healthcare models in Europe and longitudinal outcomes for young people leaving CAMHS, solutions for improving transitional care in a cost-effective manner, training modules for clinicians, and commissioning and policy guidelines for service providers and policy makers.

**Trial registration:**

“MILESTONE study” registration: ISRCTN ISRCTN83240263 Registered 23 July 2015; ClinicalTrials.gov NCT03013595 Registered 6 January 2017.

**Electronic supplementary material:**

The online version of this article (10.1186/s12888-018-1758-z) contains supplementary material, which is available to authorized users.

## Background

The journey into adult life is a time of profound physiological, psychological and social change for young people [1, 2]. Young people are expected to take responsibility for themselves, make their own decisions and become financially independent [3]. Late adolescence is also a high risk period for the emergence of mental disorders, alcohol and substance abuse and risk taking behaviour [1, 4, 5]. Overall rates of mental health problems in young people increase with age, problems become more complex, and emerging disorders, such as psychosis and personality disorders, develop [6]. Moreover, mental disorders in adolescence predict mental health problems in adulthood [7–9]. 50% of mental health problems emerge by the age of 16 years and 75% by the age of 24 [10, 11]. Intervening early when mental disorders emerge, such as in psychosis, can reduce their severity and persistence and yield positive outcomes [12, 13]. Unsatisfactory care carries a risk of illness extension, progression and chronicity, which has multiple adverse effects, including on psychosocial functioning and self-determination [14, 15]. Yet only a small proportion of young people with mental health problems, less than one in six, access services or receive appropriate care [16–18]. All over Europe, those with persisting mental health needs usually move from Child and Adolescent Mental Health Services (CAMHS) to Adult Mental Health Services (AMHS) around the critical age of 16–18 years. However, ideological, structural, functional and organisational differences between CAMHS and AMHS hamper this transition [19–29]. The disruption of care to young people at the CAMHS-AMHS interface, and the long-term adverse effects on their health, wellbeing and potential is of concern worldwide [1, 8, 30–32].

The importance of improving young people’s transition from child-orientated to adult-orientated health services has been recognised since the early 1990s [33]. For about a decade, such transition was discussed but rarely studied [34]. In the 2000s, transitions research in paediatric services increased [35], professional consensus statements were developed [36, 37] and national policy recognising the importance of transition started to emerge [38]. During this decade the importance of youth mental health [39] and improving transitions between CAMHS and AMHS started to be highlighted [24, 40, 41]. Research on transition experiences of young people with mental health problems started to be published, from sources in the USA in the early 2000 (e.g. [42, 43]) and later in the decade from the United Kingdom (UK) [44]. The transitions of care from child and adolescent mental health services to adult mental health services (TRACK) study [28, 29] and other research show that many young people with established mental health problems, such as neurodevelopmental and conduct disorders and those with emerging mood, psychotic, personality-related or substance abuse disorders slip through the care net at the transition boundary [29, 45–48]. With insufficient support in place, many disengage from mental health services altogether only to present to adult services subsequently, with more severe and enduring mental health problems [24, 49, 50]. Such occurrences may have been prevented or better controlled had better transition arrangements been in place. Young people who undergo a planned and purposeful transition process that addresses their psychosocial and medical needs, experience an improvement in their mental health and functioning [29, 47]. Yet, due to a policy-practice gap [28], few of those who do transition from CAMHS to AMHS experience ‘optimal transition’, which has been characterised by a period of parallel care between CAMHS and AMHS, at least one transition planning meeting, adequate information transfer and continuity of care [27]. Studies carried out in the Republic of Ireland [23, 51] and France [52] suggest that problems of the same nature and magnitude at the CAMHS-AMHS interface are occurring in other European countries. This poses a major health, socioeconomic and societal challenge for the care and wellbeing of young people with mental health needs within the European Union (EU), which is exacerbated by the different mental health care service structures and provision in the member states [53–55]. The development of solutions is made harder by the lack of systematic and robust evidence on the nature and severity of transition-related problems across the differing health care contexts in Europe and on their impact upon the health and wellbeing of young people.

The transitions literature often uses words such as ‘lost’ [56, 57], ‘divide’ [41] and ‘gap’ [51] to describe what happens to young people or the care of young people at the CAMHS-AMHS interface. Different models of transitional mental health care for young people have been developed to maintain continuity of care, but recent systematic reviews show few adequately powered studies, randomised controlled trials or case-controlled studies evaluating their effectiveness [19, 58, 59]. The protocol and reciprocal agreement model of transitional care, prevalent in the United Kingdom, suffers from a policy–practice disconnection [29] and organisational differences between CAMHS and AMHS [22, 60]; transition programme models, more prevalent in the United States, tend to be difficult to roll out state-wide and have not been attempted nation-wide [61–63]. No studies have evaluated the shared management framework model [57]. It is also not clear how much of the published research was informed by patient and public involvement at the design stage, rather than research studying the views of service users and their parents/carers. The quality of research into the ethical aspects of mental health transitions is poor in general [58] and the ethics of assuming transition to adult services is the ideal has been questioned by reviewers of papers and researchers because of possible risks associated with pathologising transient and self-limiting distress and dysfunction, which may be normal during adolescence.

The five year (2014–19) European Union-funded Managing the Link and Strengthening Transition from Child to Adult Mental Health Care (MILESTONE) project (grant number 602442) will create a rich evidence base on transitional mental health care in Europe. Work is subdivided into several high-quality work packages focusing on different key aspects of research, bringing together a European consortium of researchers.

### Objectives of MILESTONE

The overall aim of MILESTONE is to study transition from CAMHS to AMHS within the EU and to strengthen transitional care across different healthcare systems. The key strategic objectives are to:

1. Delineate the CAMHS-AMHS interface across all EU nation states in terms of transition of care, service organisation, legal and policy imperatives, professional training and user/carer experience.
2. Understand the processes, outcomes and experiences of transition from CAMHS to AMHS in healthcare settings across eight countries (Belgium, Croata, France, Germany, Ireland, Italy, the Netherlands, and the UK) in the EU, using a bespoke suite of measures and to develop an ethical framework for providing appropriate care to adolescents as they move to adulthood.
3. Robustly test a model of ‘managed transition’ for its clinical and cost-effectiveness in improving health and social outcomes and transition to adult health services, as compared to treatment as usual.
4. Disseminate this evidence by developing training modules for clinicians, and commissioning and policy guidelines for service providers and policy makers; and extending knowledge about transition to mental health professionals, to service users and their families, and to society in general.

### MILESTONE research

MILESTONE comprises seven freestanding research projects, which are organised into work packages (WP) (see Additional file 1):

i) mapping the CAMHS-AMHS interface and transition in all EU states via surveys (WP1);
ii) developing two measures, the Transition Readiness and Appropriateness Measure (TRAM) and the Transition Related Outcome Measure (TROM), to aid clinicians’ decision-making and stimulate shared decision-making together with young people and their parents (WP2);
iii) tracking the journey and outcomes of young people as they move out of CAMHS in a prospective cohort study in the eight MILESTONE countries* (WP3);
iv) assessing the effectiveness of a ‘managed transition’ model based on the TRAM in a nested cluster randomised controlled trial (cRCT) within the prospective cohort* (WP4);
v) determining the cost-effectiveness of the model of ‘managed transition’* (WP5);
vi) exploring ethical aspects of transitional care via qualitative and quantitative methods (WP6);
vii) understanding and further developing professional and clinical training models in CAMHS and AMHS addressing service transition via a systematic review and surveys (WP8).

*Together, these work packages constitute the “MILESTONE study”.

The main deliverables for the EU for each work package are listed in Additional file 2.

**Fig. 1:**
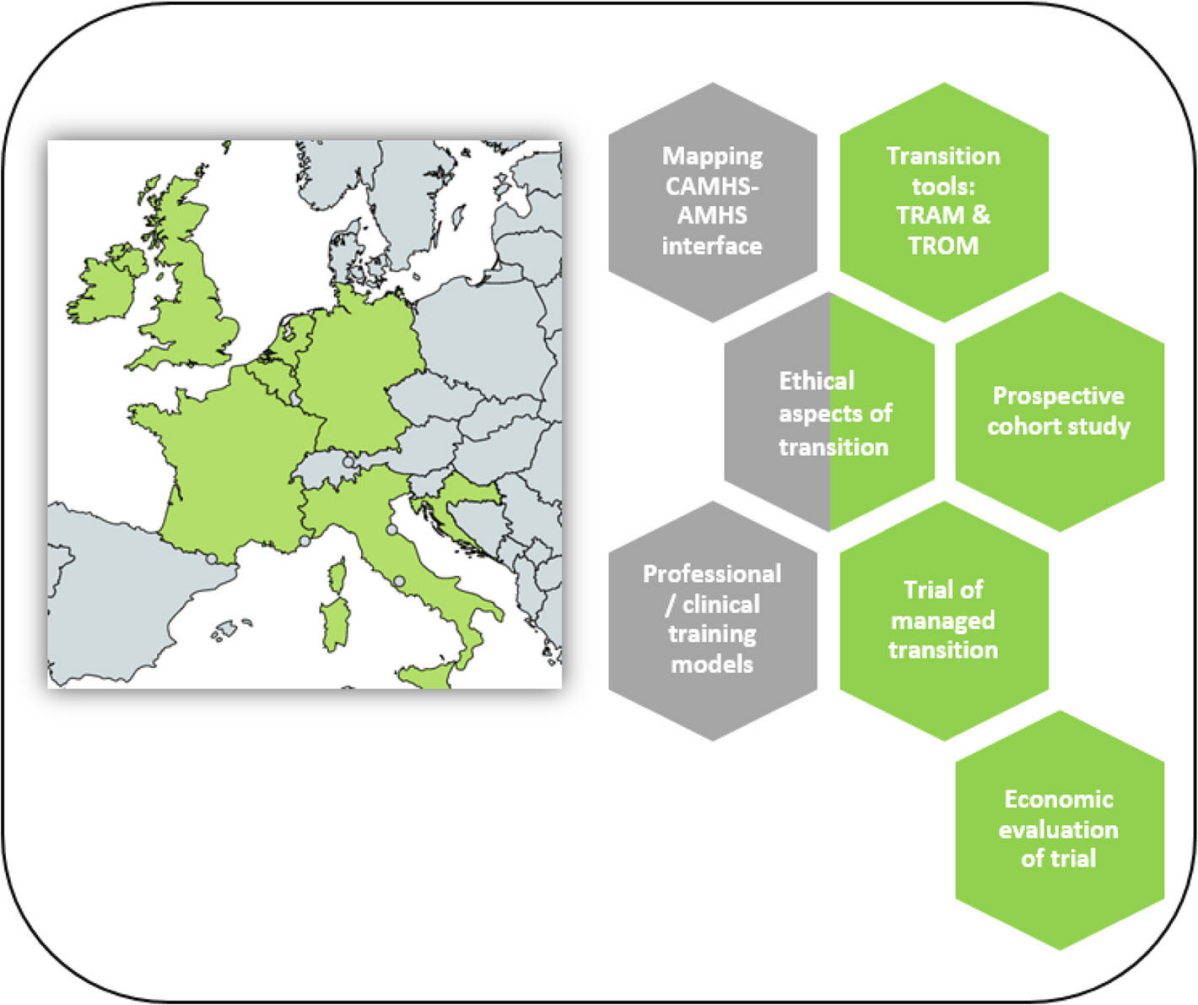
Research projects within MILESTONE. Green hexagons: research involving the eight MILESTONE countries only. Grey hexagons: research involving all European countries

Overview and geographical reach of the research projects carried out within MILESTONE are illustrated in Fig. 1.

## Methods/design

### WP 1: Mapping the CAMHS-AMHS interface across European mental health services

The aim of this work package is to analyse the clinical, organisational and legal aspects of CAMHS-AMHS interface at national and regional levels across all 28 EU states. The mapping exercise will help identify transition policies and models in different EU healthcare and social settings, and clarify how and by whom decisions about transition are made within each national mental health system. A futher aim is to examine transition in other health areas and social services in the different European countries.

A list of European governmental and non-governmental associations able to provide data on CAMHS/AMHS interface and transition was collated. Two measures were developed in English for completion online via a dedicated web domain by country experts – child psychiatrists and representatives of national child-psychiatry associations – within each of the 28 EU member states: 1) the European CAMHS Mapping Questionnaire (ECM-Q), which was based on the European Service Mapping Schedule [64] and integrated many of the domains used WHO CAMHS Atlas [65]; and 2) the Standardized Assessment Tool for Mental Health Transition (SATMEHT), which was developed from an instrument used in the TRACK study [29] and other questionnaires [34, 43, 66, 67].

For the ECM-Q, the primary aim was to characterise child and adolescent mental health care provision across countries (e.g. number of CAMHS per 100,000 young people), including collaboration with other services, activity data and funding sources. For the SATMEHT, the primary aim was to characterise the transition process and services configuration per country (e.g. proportion of young people attending CAMHS needing a transition to AMHS), including the availability of policies regulating the CAMHS-AMHS interface and the degree of stakeholders’ involvement. More detailed methodology and findings from the ECM-Q and SATMEHT have been reported elsewhere [68, 69].

### WP 2: Development and monitoring using the MILESTONE suite of measures

Ideally all young people who reach the CAMHS-AMHS transition boundary should be assessed in a structured and standardised way to determine ongoing need for care [32]. Feeding back structured assessment results to clinicians can lead to improved clinical decision making [70]. Clinical judgements made under time and resource constraints are affected by diagnostic and cognitive biases, assumptions based on patient background, a disregard of conflicting information, and misperceptions of other/adult services [70, 71]. So far a planned, purposeful and needs-based assessment of those who reach the transition boundary does not exist. Furthermore, there are no validated and reliable measures that specifically assess the experience, outcomes, and effectiveness of mental health transitional care.

The aim of this work package was therefore to create a low-burden, reliable and efficient suite of measures related to transitioning from CAMHS to AMHS, one for assessing the young person’s readiness and appropriateness for transition, and the other for measuring outcomes linked with transition. The Transition Readiness and Appropriateness Measure (TRAM) is a clinician support and assessment tool designed to identify high-risk, high-need young people for whom transition to AMHS is critical. The Transition Related Outcome Measure (TROM) assesses the quality and outcome of transition, and includes most domains present in the TRAM, allowing for comparison of results over time. Most questions in the TRAM and TROM are asked of all respondent types (young person, parent/carer and clinician); some are relevant only to the clinician and others only to the young person and parent/carer. The TRAM forms the basis of ‘managed transition’, as described below.

TRAM and TROM were developed using qualitative and quantitative methods following the US FDA Guidance for Patient-reported Outcome Measures [72]. Literature was critically reviewed for item/concept identification, and three waves of focus groups on item/concept elicitation were carried out involving young people with experience of CAMHS, their parents/carers and mental health professionals at two mental health NHS trusts in the UK. Based on the analysis of the data thus obtained, items were formed into scales and subjected to user testing so that problems with the format and completion issues could be identified. The final versions were translated into French, Italian, German, Dutch and Croatian. Participant-optimised web-based versions were developed using HealthTracker™ (https://www.healthtracker.co.uk/), a web-based portal which allows measures to be completed remotely and which has been used in other EU FP7 projects [73]. The construct validity, content validity, inter-rater reliability, test-retest reliability, and sensitivity to change of TRAM and TROM were assessed in a sub-study conducted in the eight MILESTONE countries, involving young people with experience of CAMHS, parents/carers/spouses, and mental health professionals. The development, psychometric testing and implementation of TRAM and TROM will be presented in separate publications.

The “TRAM score summary report” brings together the TRAM scores from the young person, parent/carer and CAMHS clinician with graphs visualising differences or similarities in responses. The report, which is designed as a decision support tool, contains items that are relevant to clinicians’ transition decisions (symptoms, risk factors and disruption experienced by the young person) and those that can facilitate a smooth transition. It is intended as a quick and efficient method of displaying all information relevant to transition decisions in a user-friendly, relevant and accessible format, allowing key facts to be easily transferred to care plans and referrals. The report forms the basis of ‘managed transition’ and its ease of use will be verified by questioning clinicians partaking in the MILESTONE study (see below).

### WP 3 & 4: The MILESTONE study: A longitudinal cohort study of transition of care from CAMHS to AMHS and a nested cluster randomised control trial (cRCT) of managed transition in improving outcomes for young people

We do not know the longitudinal outcomes and experiences of young people who reach the transition boundary for CAMHS in different EU countries, with varying service structures, transition ages, service provision and care. Furthermore, despite the intuitive simplicity and clinical importance of a care pathway which incorporates an evidence-based decision-making process for identifying those with on-going care need, a transition model including such an approach is not available, although its need has been articulated [74, 75].

We developed the ‘MILESTONE study’ to capture this missing information [76]. In this ongoing study we recruit and prospectively follow a large cohort of over 1000 young people approaching the CAMHS-AMHS transition boundary in the participating EU countries. The cohort study aims to evaluate the young people’s mental health, quality of life and functioning while still at CAMHS, and identify predictors of transitional trajectories, experiences and mental health outcomes over a follow-up period of two years. Nested within this cohort study is a cluster randomised controlled trial (cRCT) testing whether the implementation of the model of managed transition in CAMHS at the transition boundary improves the mental health and social outcomes of young people and their transition to adult roles when they move on from CAMHS, as compared with usual care. ‘Managed transition’ includes feedback to clinicians from the TRAM assessment. In the intervention arm clusters, clinicians are provided with TRAM Score summary reports for the young people participating in the study. Clinicians are advised to discuss the report with the young person and parent/carer, include relevant points in a transitional care plan, and to attach it to the referral letter, if further care is indicated. Young people in the control clusters receive treatment as usual, which depends on service and may or may not include transition planning.

The cohort study and cRCT share recruitment and data collection. Detailed information about eligibility criteria and methodology have been described elsewhere [76]. The primary outcome for the trial is mental health and social functioning status as measured by the Health of the Nation Outcome Scale for Child and Adolescents (HoNOSCA) at 15 months after baseline [77–79]. The measure is completed by a trained MILESTONE research assistant by interviewing the young person and taking into account all other available sources of information (parent/carer, relevant clinician and the medical records) to ensure accuracy of data [80]. The secondary outcomes are detailed in the study protocol, and include transition outcomes (TROM), self-reported and parent/carer reported psychopathology, emotional and behaviour problems of the service user (reported by both parent/carer and the young person him/herself), illness severity, quality of life, independent behaviour, illness perception, barriers to care, transition experience and readiness, and adult functioning [76]. Data collection is the same in the intervention and control clusters. Outcomes are measured 9 months (T2), 15 months (T3) and 24 months (T4) after baseline (T1).

### WP 5: Economic evaluation of the ‘managed transition’ intervention

This work package evaluates the cost-effectiveness of the model of ‘managed transition’. The aim is to assess whether the intervention conveys any benefits regarding participant health-related quality of life and HoNOSCA scores, as well as health care usage, social care usage/social costs and intervention costs as compared to treatment as usual. Data collection is embedded in the MILESTONE study [76].

Quality adjusted life years (QALYs) and HoNOSCA score are the two primary outcomes for the economic evaluation [77, 78]. The EQ-5D-5L [81] will be used to measure Health-related quality of life (HRQL). Young people in the MILESTONE study compete the measure at all four time points. Index scores [82] will be applied to calculate QALYs to ascertain the impact of the intervention on HRQL. The cost-effectiveness of the intervention on mental health will be estimated by examining changes in QALYs and HONOSCA score between the two trial arms in conjunction with the costs. The influence of alternative service delivery models and national settings on cost-effectiveness will be explored.

Health and social care resource utilisation is the secondary outcome. The MILESTONE specific Client Service Receipt Inventory (CSRI) completed at the four time points has been designed to help estimate the utilisation of resources. It draws on a CSRI used previously to estimate mental health care costs in the UK [83], but was substantially revised for use in MILESTONE. Furthermore, CAMHS/AMHS in intervention sites are asked for specific details of the impact of the ‘managed transition’ intervention in terms of the number of staff involved in transition, their workload, and additional service resources required.

### WP 6: Ethics of transitioning

An enquiring ethical stance is needed in the face of an automatic assumption that transfer of care from child to adult services is necessarily ‘good’ or appropriate in all cases [84–86]. Yet there is very little research on ethical aspects of transitional care.

The aim of this work package is therefore to a) scrutinise the assumption that transition from CAMHS to AMHS is always the best option, and b) explore the ethical/legal challenges of ensuring delivery of transitional care to those who need it most against the risk of pathologising transient and self-limiting distress and dysfunction, which may be normal during adolescence. The work package has three main parts to it: a systematic review, a focus groups with members of youth advocacy and mental health advocacy groups, and focus groups with participants of the MILESTONE study with different transition experiences. The first two parts contribute to the development of an Ethics of Transitioning questionnaire.

The systematic review focuses on ethical aspects of transitional care between child-orientated and adult-orientated health services in general; a more detailed methodology and findings have been reported elsewhere [87].

The focus groups with members of youth advocacy and mental health advocacy groups were carried out in the Republic of Ireland, the UK and Croatia by addressing ethical issues raised in vignettes. Each vignette described a young person with a particular diagnosis and mental health history approaching the end of their care at CAMHS, with a decision needed to be made regarding their onward mental health treatment. The Ethics of Transitioning questionnaire developed based on the findings of the focus groups and systematic review was included in the assessment battery of the MILESTONE study to retrieve young people’s views about ethical aspects of transitioning from all eight countries.

The focus groups with young people and parents/carers who have participated in the MILESTONE study will be conducted during the last assessment time point of the MILESTONE study in at least the three above countries. The aim will be to explore the actual experiences of the young people regarding services at the transition boundary, but also their views regarding the ethical aspects of service provision. Separate focus groups with a maximum of five participants will be held for young people and parent/carers, with a total of approximately 20 young people and 10 parent/carers taking part per country.

Data from the separate arms of the ethics work package will be integrated during the analysis phase, and linked up with findings from the MILESTONE study.

### WP 8: Training of professionals for improving transitional care across the EU

Many of the profound clinical, conceptual and ideological differences between child and adult mental health service models that contribute to transitional problems are related to psychiatry and other associated professional training. For example, the CAMHS-AMHS separation and consistent differences in care philosophies has allowed clinical focus to shift away from developmental psychiatry [19].

The aims of this work pachage are to describe current training models used across EU for CAMHS and AMHS and to assess their contribution and impact on the organisation and efficacy of transitional care at the service provider level. This will be achieved by conducting a) a systematic review on adult and child/adolescent psychiatry training and b) surveys on psychiatry and psychology training. We will also develop training models and guidelines for universities and policy makers according to the results of the studies in MILESTONE in order to optimize transition between child and adult mental health services.

The systematic review focuses on two key themes: 1) the structure and content of current training across Europe in general and adult psychiatry (GAP) and child and adolescent psychiatry (CAP) as defined by the European Union of Medical Specialists (UEMS) [88]; and 2) if and how transition is addressed in any of the GAP and CAP training. The review covers all European countries.

Bespoke questionnaires have been developed for the surveys: The psychiatry questionnaire covers adult psychiatry and child and adolescent psychiartry training in Europe, with questions for both specialties on theoretical and practical training, and transition as a subject. The questionnaire is aimed at representatives in charge of specialized training in psychiatry in all EU-countries. A similar questionnaire on psychology training aimed at representatives of national psychological associations in the eight MILESTONE countries has also been developed and circulated. A third survey focuses on trainees’ perspectives and is conducted in collaboration with the European Federation of Psychiatric Trainees.

In the analysis phase, findings will be compared and integrated with the other studies running under the MILESTONE project, for the development of a) a training programme for transitional care and b) guidelines for harmonizing CAMHS and AMHS training across the EU.

### Governance, oversight committees and patient and public involvement (PPI)

The MILESTONE consortium is a partnership between academics and clinicians from child and adult psychiatry and psychology, researchers, voluntary services and advocacy groups in eight countries. Management is through a monthly steering committee (SC, i.e. trial management group), comprising principal investigators from each MILESTONE country and core research staff. Practicalities of the MILESTONE study are discussed at regular research assistant (RA) teleconferences. The SC meets face-to-face every six months, and the whole research team annually (General Assembly). Project management is provided by concentris, a small and medium-sized enterprise specialised in the management of EU-funded projects. The conduct and progress of MILESTONE as a whole is overseen by the independent MILESTONE Scientific, Clinical and Ethical Advisory Board (SCEAB). It meets annually at the General Assembly and comprises four international experts and four Patient and Public Involvement (PPI) representatives who offer advice and monitor the progress of the project towards its stated aims.

MILESTONE has strong PPI embedded throughout the project. Initially, five British service user representatives were identified, engaged, and appropriately trained and supported to participate in the project as consultants and steering group members. They have provided advice on the development and refinement of the research methodology of the MILESTONE study, tested the suite of study documents, and recommended strategies for recruitment and retention of participants. They also assist the various other research projects running under MILESTONE, attend the SC and annual general meetings, and help plan and develop public engagement activities. In the last two years, young service users from another MILESTONE country, and carer representatives, will be involved. The ongoing work of the PPI representatives  was recognised in the UK by a service user and carer involvement in mental health  research award in 2016.

### Dissemination

Effective and creative dissemination of the MILESTONE project will be ensured throughout all its stages, from inception and recruitment to study results and recommendations to all stakeholders, including service providers, commissioners, policy makers, user and carer groups and any other target groups working at the interface between adolescence and adult mental health care. To date, this includes a TEDx talk and a film on the theme of transition developed through participatory workshops involving MILESTONE PPI representatives and researchers.

## Discussion

The MILESTONE project is to the best of our knowledge the first of its kind in the scope and scale of research focused specifically on transitions at the CAMHS-AMHS interface across Europe. It will provide a comprehensive yet nuanced account of the organisation, policy, and practice of care for young people with mental health needs at the CAMHS-AMHS boundary across the EU, and a timely analysis of their outcomes and experiences. This will help identify opportunities to improve their health care outcomes, social functioning, and quality of life, enabling them to more easily progress to meaningful adult roles.

Although there are some existing service level innovations in Australia (ORYGEN, Melbourne https://oyh.org.au/), Ireland (JIGSAW, https://www.jigsaw.ie/) and UK (Forward Thinking Birmingham, https://www.forwardthinkingbirmingham.org.uk/) that are attempting, in their different healthcare contexts, to redefine service structures for young people with mental health needs (up to 25 years), there is still much to be done. The practice of having a CAMHS-AMHS divide is deeply rooted in European mental health provision and tradition. First politically relevant steps are being taken in some countries. In Germany, for example, national specialist societies are identifying the need for interdisciplinary cooperation and a “Transitional Psychiatry Task Force” has been created. It is expected that the findings of MILESTONE will encourage services across Europe to question their structure and identify current weaknesses in the CAMHS-AMHS care pathway so that outcomes for young people and their families can be improved by adapting best evidence-based practice into their service provision. Proposed changes to services should ensure that those young people who need it receive on-going care and that others with transient or remitted conditions are not ‘pathologised’ and do not receive inappropriate, unnecessary or potential harmful interventions.

Although structural weaknesses at the CAMHS-AMHS interface have been recognised for almost 25 years [33], the evidence base is still weak and the need for high quality research and common efforts at different levels (across countries, involving various research organisations, professional bodies and specialist societies) remains high. Action is needed to bring together the seemingly disparate worlds of child/adolescent and adult mental health services and learn from other countries about how to minimise policy-practice gaps, and improve transition procedures and outcomes. Any research should be informed by and involve young people who have experienced transition from CAMHS [89].

## Additional files

Additional file 1: MILESTONE work packages (WP), WP leaders and partner institutions. The table contains information about work package titles, names of the work package leaders, their institutions, and countries. (DOCX 13 kb).

Additional file 2: MILESTONE work package deliverables for the EU. The table contains information about the deliverables linked with each workpackage that have to be submitted to the EU. (DOCX 20 kb).

## Abbreviations

AMHS: Adult Mental Health Services
CAMHS: Child and Adolescent Mental Health Services
CI: Chief Investigator
cRCT: Cluster Randomised Controlled Trial
CSRI: Client Service Receipt Inventory
EQ-5D-5L: Euroquol (quality of life measure)
EU: European Union
HoNOSCA: Health of the Nation Outcome Scale for Children and Adolescents
HRQL: Health-related quality of life
ISRCTN: International Standard Randomised Controlled Trial Number
PI: Principal Investigator
PPI: Patient and Public Involvement
QALY: Quality adjusted life year
RA: Research Assistant
SCEAB: Scientific Clinical and Ethical Advisory Board
TB: Transition Boundary
TRAM: Transition Readiness and Appropriateness Measure
TROM: Transition Related Outcome Measure
YP: Young person
