# Correction to: Managing the link and strengthening transition from child to adult mental health Care in Europe (MILESTONE): background, rationale and methodology

**DOI:** 10.1186/s12888-018-1881-x

**Published:** 2018-09-14

**Authors:** H. Tuomainen, U. Schulze, J. Warwick, M. Paul, G. C. Dieleman, T. Franić, G. de Girolamo, J. Madan, A. Maras, F. McNicholas, D. Purper-Ouakil, P. Santosh, G. Signorini, C. Street, S. Tremmery, F. C. Verhulst, D. Wolke, S. P. Singh, for the MILESTONE consortium

**Affiliations:** 1 Mental Health and Wellbeing, Division of Health Sciences, Warwick Medical School, University of Warwick, Coventry, UK; 2 Department of Child and Adolescent Psychiatry/Psychotherapy, University of Ulm, Ulm, Germany; 3 Warwick Clinical Trials Unit, Warwick Medical School, University of Warwick, Coventry, UK; 4 Coventry and Warwickshire Partnership NHS Trust, Coventry, UK; 5 Department of Child and Adolescent Psychiatry and Psychology, Erasmus Medical Center Rotterdam, Rotterdam, Netherlands; 6 Department of Psychiatry, Clinical Hospital Center Split, Split, Croatia; 7 Warwick Medical School, University of Warwick, Coventry, UK; 8 Yulius Academy, Yulius Mental Health Organization, Barendrecht, Netherlands; 9 Department of Child and Adolescent Psychiatry, University College Dublin School of Medicine and Medical Science, Dublin, Republic of Ireland; 10 Geary Institute, University College Dublin, Dublin, Republic of Ireland; 11 Centre Hospitalier Universitaire de Montpellier, Montpellier, France; 12 Department of Child and Adolescent Psychiatry, Institute of Psychiatry, Psychology and Neuroscience, King’s College London, London, UK; 13 HealthTracker Ltd, Gillingham, UK; 14 Psychiatric Epidemiology and Evaluation Unit, Saint John of God Clinical Research Center, Brescia, Italy; 15 Department of Neurosciences, Child & Adolescent Psychiatry, University of Leuven, Leuven, Belgium; 16 Department of Child & Adolescent Psychiatry, University Hospitals Leuven, Leuven, Belgium; 17 Department of Psychology, University of Warwick, Coventry, UK; 18 Department of Child Psychiatry, Our Lady’s Hospital for Sick Children, Dublin, Republic of Ireland; 19 Lucena Clinic SJOG, Dublin, Republic of Ireland; 20 Centre for Interventional Paediatric Psychopharmacology and Rare Diseases (CIPPRD), National and Specialist Child and Adolescent Mental Health Services, Maudsley Hospital, London, UK

## Correction

Following publication of the original article [1], the authors reported they wanted to reinstate a co-author, who previously declined his authorship due to a misinterpretation of authorship limitations per research center. In this Correction, Dr. de Girolamo is included as co-author on the paper. Dr. de Girolamo has contributed to the design and conduct of the MILESTONE project.
