# Supplementary material for: Managing the link and strengthening transition from child to adult mental health Care in Europe (MILESTONE): background, rationale and methodology
Source: BMC Psychiatry. 2018 Jun 4;18:167. doi: 10.1186/s12888-018-1758-z (PMC5987458; doi:10.1186/s12888-018-1758-z)
Supplement: Supplementary file 1 — MILESTONE work packages (WP), WP leaders and partner institutions. The table contains information about work package titles, names of the work package leaders, their institutions, and countries. (DOCX 13 kb). [file 12888_2018_1758_MOESM1_ESM.docx]

MILESTONE work packages (WP), WP leaders and partner institutions.

| **WORK PACKAGES** | **WP LEADER** | **Institution/s** | **COUNTRY** |
| --- | --- | --- | --- |
| **WP1** Mapping the CAMHS-AMHS Interface across European Mental Health Services | Dr. Giovanni de Girolamo | IRCCS – Fatebenefratelli | Italy |
| **WP2** Development and monitoring using the MILESTONE Suite of Measures | Prof. Paramala Santosh | King’s College London | United Kingdom |
| **WP3** Longitudinal cohort study of transition of care from CAMHS to AMHS | Dr. Athanasios Maras  Prof. Frank Verhulst  Dr. Gwen Dieleman | Yulius Academy, Yulius Mental Health Organisation  Erasmus Medical Centre | The Netherlands |
| **WP4** Cluster-randomised control trial of managed transition in improving outcomes for young people | Prof. Swaran Singh | University of Warwick | United Kingdom |
| **WP5** Economic Evaluation of the Experimental Intervention | Dr. Jason Madan | University of Warwick | United Kingdom |
| **WP6** Ethics | Prof. Fiona McNicholas | University College Dublin | Ireland |
| **WP7** Dissemination of Study Progress and Results to Key Stakeholders | Dr. Ulrike Schulze  Prof. Tomislav Franić | University of Ulm  Klinički bolnički centar Split | Germany  Croatia |
| **WP8** Training programme for improving transitional care across the EU | Prof. Diane Purper-Ouakil  Prof. Sabine Tremmery | Centre Hospitalier Universitaire de Montpellier  Katholieke Universiteit Leuven | France  Belgium |
| **WP9** Project Management | Andrea Wohner | concentris research management gmbh | Germany |
