# Supplementary material for: Managing the link and strengthening transition from child to adult mental health Care in Europe (MILESTONE): background, rationale and methodology
Source: BMC Psychiatry. 2018 Jun 4;18:167. doi: 10.1186/s12888-018-1758-z (PMC5987458; doi:10.1186/s12888-018-1758-z)
Supplement: Supplementary file 2 — MILESTONE work package deliverables for the EU. The table contains information about the deliverables linked with each workpackage that have to be submitted to the EU. (DOCX 20 kb). [file 12888_2018_1758_MOESM2_ESM.docx]

**MILESTONE work package deliverables for the EU**

| **WP01** | **Mapping the CAMHS-AMHS Interface across European Mental Health Services** | |
| --- | --- | --- |
| D1.1 | Standardized Assessment Tool for Mental Health Transition (SATMEHT) | Standardized Assessment Tool for Mental Health Transition (SATMEHT): This will be a standardized instrument to assess the main service characteristics of transition for adolescents in treatment for (i) any mental disorders or (ii) any alcohol or substance abuse disorder. It will include items covering the main characteristics of service provision in the country (both CAMHS and AMHS, plus services for alcohol/substance abuse), data about number of services per 100,000 popn (including number of professionals), whether services have a standard policy for transition at national or regional level, how transition is organized, possibly activity data related to transition (number of patients in treatment at CAMHS in the age range 15-18, and at AMHS in the age range 17-21), data about training of professionals in the area of adolescent mental health and information about policies and services for early interventions. This Deliverable will be submitted in form of a report. |
| D1.2 | Report on Transition Pathways in European mental health services with data on the clinical, organisational and legal aspects of CAMHS-AMHS interface | Report on Transition Pathways in European mental health services with data on the clinical, organisational and legal aspects of CAMHS-AMHS interface: Report on Transition Pathways in European mental health services with data on the clinical, organisational and legal aspects of CAMHS-AMHS interface including transition policies, models and outcomes where available, and report from a two day meeting of CAMHS experts across the EU. This Deliverable will include all information gathered through the SATMEHT, plus the information obtained at the planned meeting. It will include up-to-date quantitative data about CAMHS and AMHS in the 27 European countries (e.g., number of services per 100,000 popn, number of beds per 100,000 popn, etc), characteristics and interface of CAMHS and AMHS, essential of mental health policies (national vs regional standards), procedures and models identified, categorisation of the services, how decisions are made on transition. It will also include names of key people at national level in the area of mental health, both at governmental and professional levels. |
|  |  |  |
| **WP02** | **Development and monitoring using the MILESTONE Suite of Measures** | |
| D2.1 | Development and translation of MILESTONE Suite of Instruments | Development and translation of MILESTONE Suite of Instruments (including TROM and TRAM): The newly developed instruments will be translated into the languages necessary in the different countries represented in the consortium. |
| D2.2 | Development of web-based versions of the MILESTONE Suite of instruments on HealthTracker platform | Development of web-based versions of the MILESTONE Suite of instruments on HealthTracker platform: All the measures will be modified to suit web-based presentations on HealthTracker so that they can be used in WP3 and 4. |
| D2.3 | Report on the results of the development procedure of the MILESTONE Suite of Measures and methodology of validation | Report on the results of the development procedure of the MILESTONE Suite of Measures and methodology of validation: The validation of the MILESTONE Suite of measures will be published based on the results from the participating sites after the completion of WP3 and 4. In addition to the report, a publication will be submitted to an international, peer-reviewed journal. |
| D2.4 | Report on the process and delivery of data extraction, monitoring support and optimisation of TRAM on HealthTracker^TM^ | Report on the process and delivery of data extraction, monitoring support and optimisation of TRAM on  HealthTracker: Report on the process and delivery of data extraction, monitoring support and optimisation of  TRAM on HealthTracker. |
|  |  |  |
| **WP03** | **Longitudinal cohort study of transition of care from CAMHS to AMHS** | |
| D3.1 | Report on mental health outcomes of service users for whom CAMHS ends | Report on mental health outcomes of service users for whom CAMHS ends: Description of the characteristics of adolescent patients for whom CAMHS ends; i.e. diagnostic status in terms of DSM-IV(V) and ICD-10 diagnoses, adolescents' needs for care, emotional and behavioural problems, quality of life, global functioning, socio-demographic, social functioning, academic functioning, mental health and physical health. In addition to the report, a publication will be submitted to an international, peer-reviewed journal. |
| D3.2 | Report on mental health and general functioning of adolescents who reach the CAMHS transition boundary across the transition into young adulthood | Report on mental health and general functioning of adolescents who reach the CAMHS transition boundary across the transition into young adulthood: Description of the longitudinal course of self-reported need for care, self- and parent reported emotional and behavioural problems, use of mental health in adolescents who cross the CAMHS transition boundary during an eighteen-month-follow up period. In addition to the report, a publication will be submitted to an international, peer-reviewed journal. |
| D3.3 | Report on outcomes of those service users for whom CAMH ends who transit to AMHS versus those who do not | Report on outcomes of those service users for whom CAMH ends who transit to AMHS versus those who do not: Comparison of diagnostic status (DSM-IV(V) and ICD-10), emotional and behavioural problems, adolescents' needs for care, emotional and behavioural problems, quality of life, global functioning, socio-demographic, social functioning, academic functioning, mental health and physical health between adolescents for whom CAMHS ends who make the transition versus those who do not make the transition. In addition to the report, a publication will be submitted to an international, peer-reviewed journal. |
|  |  |  |
| **WP04** | **Cluster- randomised control trial of managed transition in improving outcomes for young people who reach the CAMHS – AMHS transition boundary** | |
| D4.1 | Final version of trial protocol as submitted to regulators/EC | Final version of trial protocol as submitted to regulators/EC: The trial protocol will be a written procedural method that will describe in detail the experimental intervention (Managed Transition). It will include background information about the trial, the objectives and purpose of the trial, trial design including randomisation, inclusion and exclusion criteria, the intervention, procedures to ensure fidelity, primary and secondary endpoints, duration, discontinuation rules. Safety parameters, SOPs for risk assessment, risk management and research governance and the procedures for quality assurance. |
| D4.2 | Registration number of CT in a WHO- or ICMJE-approved registry | Registration number of CT in a WHO- or ICMJE-approved registry: We will register the trial in a registry that meets the criteria set by the International Committee of Medical journal Editors (ICJME). These criteria state that registry must be accessible to the public at no charge, open to all potential participants, managed by a not-for-profit organisation, have mechanisms to ensure the validity of the registration data and should be electronically accessible. Once registered, our trial will get a unique identifier which we will submit to the EU. |
| D4.3 | Report on status of the posting of results (to the trial registry) | Report on status of the posting of results (to the trial registry): Report on the submission of the trial data online. |
| D4.4 | Report on trial findings | Report on trial findings: We will submit the trial findings as a scientific paper to a high quality peer  reviewed international journal such as the Lancet or the BMJ. A report on the trial findings will be sent to the  EC, separately. |
|  |  |  |
| **WP05** | **Economic Evaluation of the Managed Transition Intervention** | |
| D5.1 | Publication on cost-effectiveness analysis submitted to an international, peer reviewed journal | Report on cost-effectiveness analysis submitted to an international, peer reviewed journal: A report drafted in a format suitable for publication in an international peer-reviewed academic journal, describing the methods, results and conclusions of the economic evaluation carried out alongside the clinical trial. |
|  |  |  |
| **WP06** | **Ethics** | |
| D6.1 | Submission of three SOPs | Submission of three SOPs: SOP I: "Informed consent, assent and determination of capacity"; SOP II "Participant privacy and confidentiality in the MILESTONE Study"; SOP III: "SOP Response to Adverse Situations in the MILESTONE Study". |
| D6.2 | Ethics and regulatory approvals for the studies | Ethics and regulatory approvals for the studies: Ethical approval will be sought and received and available for review for each aspect of the study in all participating countries in the study. This includes the registration, protocol and ethics approval by the competent legal local/national Ethics Boards/Bodies/ administrations. Any necessary amendments to the study design will also be submitted before the relevant ethics committees and appropriate documentation securely held. |
| D6.3 | A review of the literature on ethical aspects of transitional care | A review of the literature on ethical aspects of transitional care: The authors will conduct a systematic review on the ethical issues involved in aspects of transition and transfer of care from one service to another, with particular emphasis on mental health services. Key words include ethic*, transfer, transition, MH and psychological services. |
| D6.4 | Report on service user and carer involvement in the MILESTONE Project | Report on service user and carer involvement in MILESTONE: The study team will write a report documenting the contributions and experiences of service users and carers during the conduct of the study. This should inform other investigators conducting research with young people and their carers and contribute to real and valued participation of individuals with mental health difficulties. |
| D6.5 | Report on ethical challenges of ensuring delivery of transitional care (findings from both focus groups ) | Report on ethical challenges of ensuring delivery of transitional care (findings from focus groups): Findings from focus groups carried out with young people with enduring mental health difficulties, their parents and voluntary providers and charities will be presented in a report. This will highlight the positive and negative aspects linked with transfer or termination of care, transition to another services and the attitudes, barriers and enablers around this process. |
|  |  |  |
| **WP07** | **Dissemination of Study Progress and Results to Key Stakeholders** | |
| D7.1 | Project website public part online | Project website public part online: The public part of the project website shall comprise the most important facts explaining the MILESTONE project. The target group of the website shall be researchers, interested laymen, persons concerned and stakeholders. The contents of the website will be designed accordingly to the needs of each target group. The website will be updated on a regular basis so that it remains attractive for all target groups. A report will be submitted on the website. |
| D7.2 | Report on the dissemination plan | Report on the dissemination plan: The dissemination plan will comprise several steps to inform politicians and key stakeholders about the project and its main results - with the intention to involve them with the help of concrete suggestions and to keep them motivated. |
| D7.3 | Presentation of project and its findings at national and international scientific conferences | Presentation of project and its findings at national and international scientific conferences: A presentation of the project will be ready in Month 12. This presentation shall serve as a basis for national and international conferences and will be uploaded on the website. At the end of the project (Month 55), a presentation of all project findings will be created and published on the public part of the MILESTONE website. The presentations will be sent to the EC. |
|  |  |  |
| **WP08** | **Training programme for improving transitional care across the EU** | |
| D8.1 | Report on investigator training (session 1) | Report on investigator training (session 1): This report will also include an evaluation of the session. |
| D8.2 | Report on investigator training (session 2) | Report on investigator training (session 2): This report will also include an evaluation of the sessions. |
| D8.3 | Report on training programme and guidelines | Report on training programme and guidelines: In addition to the report, a publication will be submitted to  an international, peer-reviewed journal |
